# Author Correction: Homology directed telomere clustering, ultrabright telomere formation and nuclear envelope rupture in cells lacking TRF2^B^ and RAP1

**DOI:** 10.1038/s41467-023-39144-7

**Published:** 2023-06-07

**Authors:** Rekha Rai, Kevin Biju, Wenqi Sun, Tori Sodeinde, Amer Al-Hiyasat, Jaida Morgan, Xianwen Ye, Xueqing Li, Yong Chen, Sandy Chang

**Affiliations:** 1 Department of Laboratory Medicine, Yale University School of Medicine, New Haven, 330 Cedar Street, CT 06520 USA; 2 Johns Hopkins University School of Medicine, Baltimore, MD 21205 USA; 3 State Key Laboratory of Molecular Biology, National Center for Protein Science Shanghai, Shanghai Institute of Biochemistry and Cell Biology, Center for Excellence in Molecular Cell Science, Chinese Academy of Sciences, Shanghai, 200031 China; 4 University of Chinese Academy of Sciences, 100049 Beijing, China; 5 School of Life Science and Technology, ShanghaiTech University, 100 Haike Road, Shanghai, 201210 China; 6 Department of Pathology, Yale University School of Medicine, 330 Cedar Street, New Haven, CT 06520 USA; 7 Department of Molecular Biophysics and Biochemistry, Yale University School of Medicine, 330 Cedar Street, New Haven, CT 06520 USA

**Keywords:** Homologous recombination, Double-strand DNA breaks

Correction to: *Nature Communications* 10.1038/s41467-023-37761-w, published online 14 April 2023

In this article the author name Amer Al-Hiyasat was incorrectly written as Amer Al-Hiysat. The original article has been corrected.

