# Supplementary material for: Homology directed telomere clustering, ultrabright telomere formation and nuclear envelope rupture in cells lacking TRF2B and RAP1
Source: Nat Commun. 2023 Apr 14;14:2144. doi: 10.1038/s41467-023-37761-w (PMC10104862; doi:10.1038/s41467-023-37761-w)
Supplement: Supplementary file 1 — Supplementary Information [file 41467_2023_37761_MOESM1_ESM.pdf]

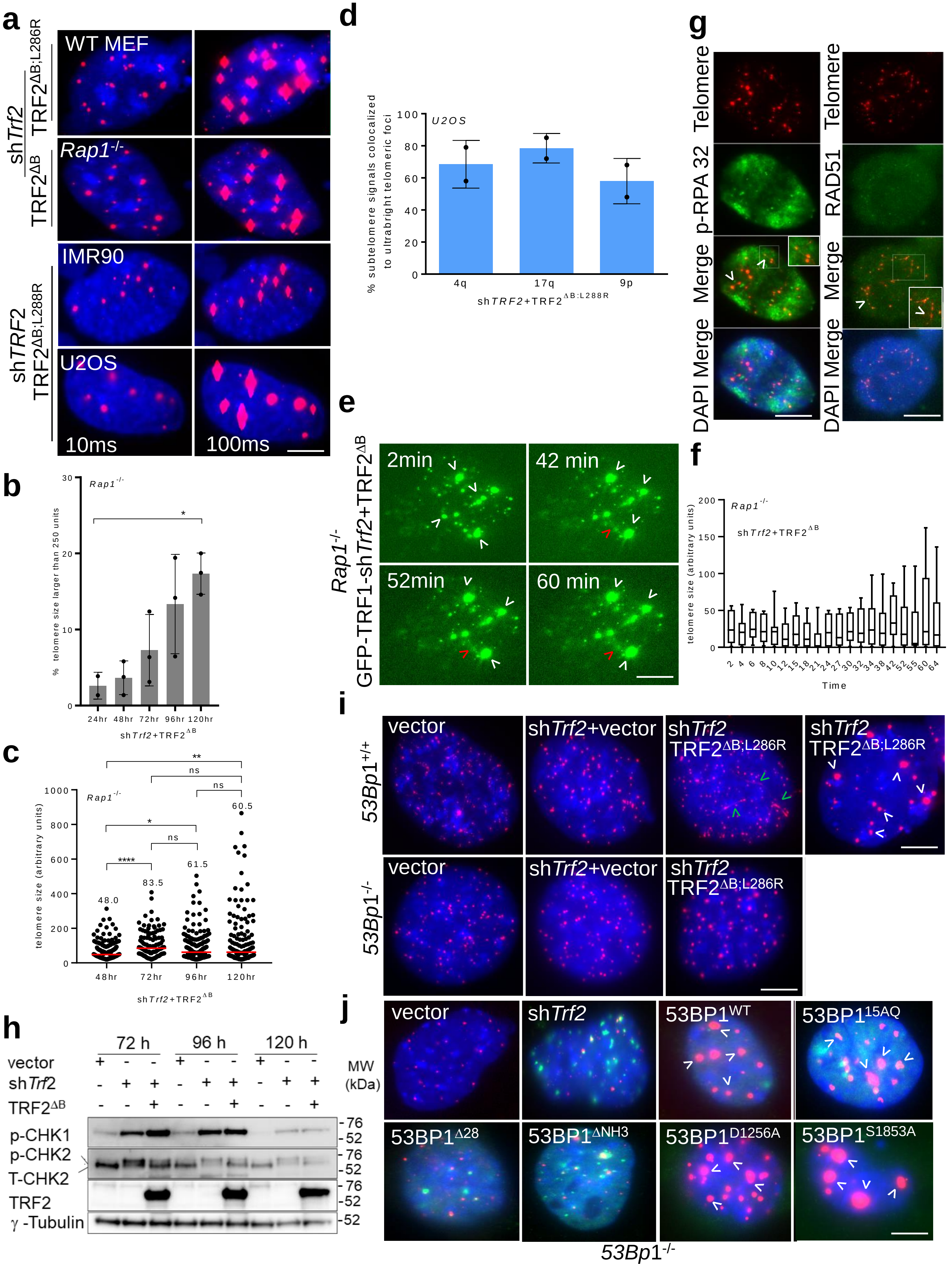

a
WT MEF
shTrf2
TRF2ΔB;L286R
Rap1-/-
TRF2ΔB
IMR90
U2OS
shTRF2
TRF2ΔB;L288R
10ms
100ms
b
Rap1-/-
% telomere size larger than 250 units
24hr
48hr
72hr
96hr
120hr
shTrf2+TRF2ΔB
c
Rap1-/-
telomere size (arbitrary units)
48.0
83.5
61.5
60.5
ns
48hr
72hr
96hr
120hr
shTrf2+TRF2ΔB
d
U2OS
% subtelomere signals colocalized to ultrabright telomeric foci
4q
17q
9p
shTRF2+TRF2ΔB:L288R
e
Rap1-/-
GFP-TRF1-shTrf2+TRF2ΔB
2min
42 min
52min
60 min
f
Rap1-/-
shTrf2+TRF2ΔB
telomere size (arbitrary units)
Time
g
Telomere
p-RPA 32
Merge
DAPI Merge
RAD51
h
72 h
96 h
120 h
vector
shTrf2
TRF2ΔB
MW (kDa)
p-CHK1
p-CHK2
T-CHK2
TRF2
γ -Tubulin
i
53Bp1+/+
53Bp1-/-
vector
shTrf2+vector
shTrf2 TRF2ΔB;L286R
j
vector
shTrf2
53BP1WT
53BP115AQ
53BP1Δ28
53BP1ΔNH3
53BP1D1256A
53BP1S1853A
53Bp1-/-

Supplementary Fig. 1 a. Representative PNA-FISH of interphase nuclei from three independent experiments reveal UT foci in U2OS, IMR90, WT and *Rap1*$^{-/-}$ MEFs expressing the indicated DNA constructs imaged at 10ms and 100ms exposures. At 100ms exposure, UTs saturate the CCD detectors, causing blooming artifacts (diamond shaped foci). Scale bars: 5µm. **b.** Quantification of UTs larger than 250 arbitrary units in *Rap1*$^{-/-}$ MEFs expressing sh*Trf2* + TRF2$^{\Delta B}$ at the indicated time. Data represents the mean of three independent experiments ± SD from a minimum 200 nuclei analyzed per experiment. *P=0.0241 by one-way ANOVA. **c**. Quantification of telomere size in *Rap1*$^{-/-}$ MEFs expressing sh*Trf2* + TRF2$^{\Delta B}$ at the indicated times from one representative experiment. At least a minimum 200 nuclei analyzed per experiment. Red line: median. *P=0.0113, **P=0.051, ****P<0.0001 by Kruskal-Wallis test with Dunn's multiple comparison. ns: non-significant. **d**. Percent UTs colocalized with chromosome specific sub telomere in U2OS cells expressing TRF2$^{\Delta B;L288R}$. Mean value ± SD from two independent experiments are shown. **e.** Live cell imaging using GFP-TRF1 as the telomere marker (green) in *Rap1*$^{-/-}$ MEFs expressing sh*Trf2* + TRF2$^{\Delta B}$ over the indicated times. Telomere bridges and UTs are shown with red and white arrowheads, respectively. Scale bars: 5µm. **f**. Quantification of telomere sizes over time due to telomere-telomere clustering in (**e**) from one representative experiment. Box show the median and interquartile range (25% to 75%). **g**. Representative image from two independent experiments showing telomeric bridges co-localized with p-RPA32 or RAD51 in *Rap1*$^{-/-}$ MEFs expressing sh*Trf2* + TRF2$^{\Delta B}$. Arrowheads point to sites of p-RPA32 or RAD51 localization on the telomere bridges. Insets show enlarged images. Scale bars: 5µm. **h**. Immunoblots for phospho (p)-CHK2 and p-CHK1 in *Rap1*$^{-/-}$ MEFs expressing TRF2$^{\Delta B}$ reveal the presence of increased single-stranded DNA mediated damage when both RAP1 and TRF2^{B} are deleted. γ-Tubulin used as loading control. The blot shown is the representative of two independent experiments. **i**. Representative PNA-FISH of interphase nuclei from two independent experiments showing CCCTAA-positive filaments (green arrowheads) and UTs (white arrowheads) in interphase nuclei of *53Bp1*$^{+/+}$ and *53Bp1*$^{-/-}$ MEFs expressing sh*Trf2* + TRF2$^{\Delta B;L286R}$. Scale bars: 5µm. **j**. Representative IF-FISH from two independent experiments for HA-tagged 53BP1 (green) in the interphase nuclei showing UT foci in *53Bp1*$^{-/-}$ reconstituted with either HA-53BP1^{WT} or indicated 53BP1 mutants in the presence of sh*Trf2* + TRF2$^{\Delta B;L286R}$. Arrowheads point to UTs. Scale bars: 5µm.

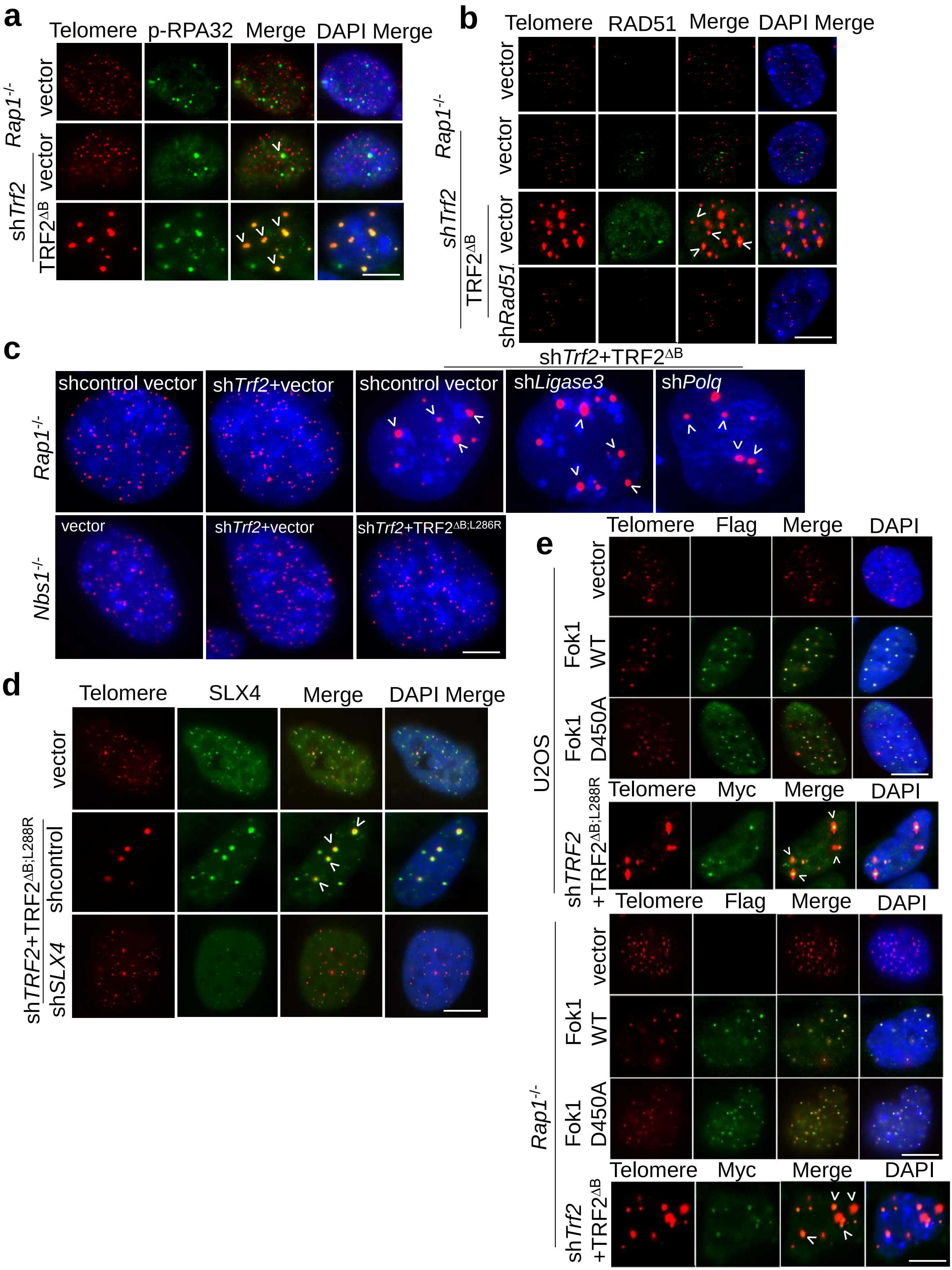

a
Telomere
p-RPA32
Merge
DAPI Merge
Rap1-/-
vector
shTrf2
vector
TRF2ΔB
b
Telomere
RAD51
Merge
DAPI Merge
vector
shRad51
c
shcontrol vector
shTrf2+vector
shTrf2+TRF2ΔB
shLigase3
shPolq
Nbs1-/-
shTrf2+TRF2ΔB;L286R
d
SLX4
shTRF2+TRF2ΔB;L288R
shcontrol
shSLX4
e
Flag
DAPI
Fok1 WT
Fok1 D450A
U2OS
Myc
shTRF2 +TRF2ΔB;L288R
shTrf2 +TRF2ΔB

Supplementary Fig. 2 a. Representative IF-FISH images from three independent experiments showing p-RPA32 (S33) (green) co-localized with UTs in *Rap1*$^{-/-}$ MEFs cells expressing TRF2$^{\Delta B}$. Scale bars: 5µm. **b**. Representative IF-FISH images from three independent experiments revealed that RAD51 (green) co-localized with UTs in *Rap1*$^{-/-}$ MEFs expressing TRF2$^{\Delta B}$. Scale bars: 5µm. **c**. Representative PNA-FISH of interphase nuclei from two independent experiments showing UTs in the absence of NBS1 and factors that promote A-NHEJ. Arrowheads point to UTs**.** Scale bars: 5µm. **d.** Representative IF-FISH images from three independent experiments showing the effect of SLX4 depletion in generation of UTs in U2OS cells treated with sh*TRF2*+TRF2$^{\Delta B;L288R}$. Scale bars: 5µm. **e**. Representative IF-FISH images from three independent experiments showing small telomere foci (arrowheads) upon expressing Flag-tagged TRF1 WT FokI and FokI^{D450A} (green) in U2OS cells (100ms exposures). Middle and bottom panels show that UTs (arrowheads) are significantly larger than FokI induced foci in U2OS and *Rap1*$^{-/-}$ MEFs expressing TRF2$^{\Delta B;L288R}$ and TRF2$^{\Delta B;L286R}$ (100 ms exposure). Scale bars: 5µm.

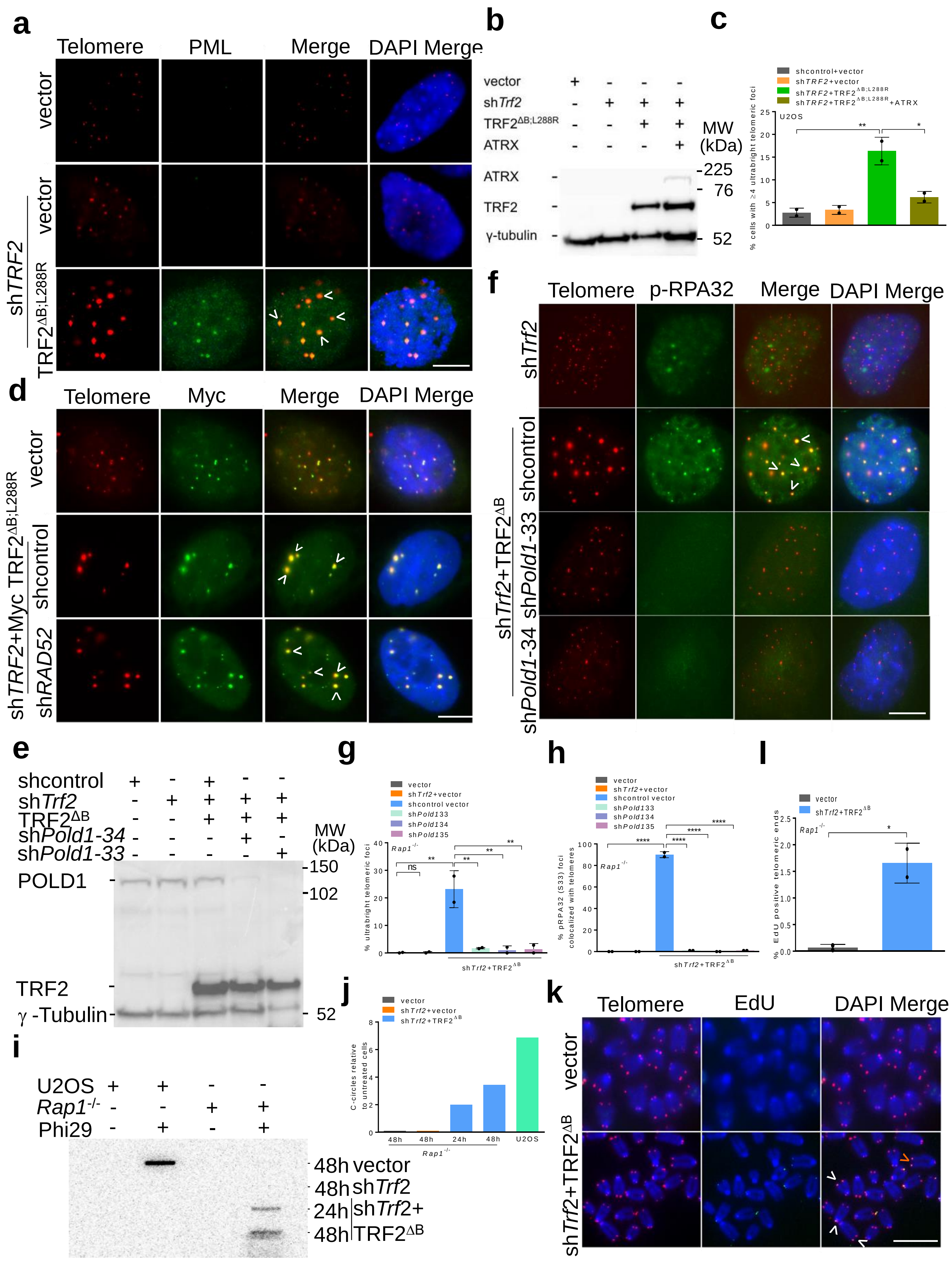

a
Telomere
PML
Merge
DAPI Merge
vector
shTRF2
vector
TRF2ΔB;L288R
b
vector
shTrf2
TRF2ΔB;L288R
ATRX
MW (kDa)
ATRX
TRF2
γ-tubulin
225
76
52
c
shcontrol+vector
shTRF2+vector
shTRF2+TRF2ΔB;L288R
shTRF2+TRF2ΔB;L288R+ATRX
U2OS
% cells with ≥4 ultrabright telomeric foci
d
Telomere
Myc
Merge
DAPI Merge
vector
shcontrol
shRAD52
shTRF2+Myc TRF2ΔB;L288R
f
Telomere
p-RPA32
Merge
DAPI Merge
shTrf2
shcontrol
shPold1-33
shPold1-34
shTrf2+TRF2ΔB
e
shcontrol
shTrf2
TRF2ΔB
shPold1-34
shPold1-33
MW (kDa)
POLD1
TRF2
γ -Tubulin
150
102
52
g
vector
shTrf2+vector
shcontrol vector
shPold133
shPold134
shPold135
Rap1-/-
% ultrabright telomeric foci
ns
shTrf2+TRF2ΔB
h
vector
shTrf2+vector
shcontrol vector
shPold133
shPold134
shPold135
Rap1-/-
% pRPA32 (S33) foci colocalized with telomeres
shTrf2+TRF2ΔB
l
vector
shTrf2+TRF2ΔB
Rap1-/-
% EdU positive telomeric ends
i
U2OS
Rap1-/-
Phi29
48h vector
48h shTrf2
24h shTrf2+
48h TRF2ΔB
j
vector
shTrf2+vector
shTrf2+TRF2ΔB
C-circles relative to untreated cells
48h
48h
24h
48h
U2OS
Rap1-/-
k
Telomere
EdU
DAPI Merge
vector
shTrf2+TRF2ΔB

Supplementary Fig. 3 a. Representative images of IF-FISH from three independent experiments showing that PML bodies (green) co-localized with UTs in U2OS cells expressing sh*TRF2*+TRF2$^{\Delta B;L288R}$. Scale bars: 5µm. **b.** Immunoblot showing the reconstitution of GFP-ATRX in U2OS cells. Anti-GFP and anti-Myc antibodies used to detect ATRX and TRF2 protein. γ-Tubulin served as loading control. The blot shown is the representative of two independent experiments. **c.** Quantification of UTs in GFP-ATRX reconstituted U2OS cells. Data shown as the mean of two experiments with ± SD from a minimum 250 nuclei analyzed per experiment. *P=0.0163, **P=0.0057 by one-way ANOVA. **d**. Representative images of IF-FISH from three independent experiments showing the effect of RAD52 depletion in the generation of UTs in U2OS cells treated with shTRF2+TRF2$^{\Delta B;L288R}$. Scale bars: 5µm. Myc antibody (green) is used to detect TRF2. **e**. Immunoblot showing knockdown efficiency of *Pold1* with three different shRNAs in *Rap1*$^{-/-}$ MEFs expressing indicated DNAs. Anti-POLD1 and TRF2 antibody used to detect POLD1 and TRF2 proteins. γ-Tubulin served as loading control. The blot shown is the representative of two independent experiments. **f**. Representative IF-FISH images showing depletion of POLD1 reduced the formation of UTs and p-RPA32 (S33) (green) TIFs in *Rap1*$^{-/-}$ cells treated with sh*Trf2*+TRF2$^{\Delta B}$. Scale bars: 5µm. **g**. Quantification of UTs in (**f**). Data represents the mean of two independent experiments ± SD from a minimum 350 nuclei analyzed per experiment. **P=0.0017 by one-way ANOVA. ns: non-significant. **h**. Quantification of percent UTs co-localized with p-RPA32 (S33) in (**f**). Data represents the mean of two independent experiments ± SD from a minimum 350 nuclei analyzed per experiment. ****P<0.0001 by one-way ANOVA. **i**. C-circle analysis using 400µg genomic DNA isolated from *Rap1*$^{-/-}$ MEFs expressing indicated DNAs in the absence and presence Phi29 DNA polymerase. 400µg genomic DNA from U2OS cells was used as positive control. **j**. Quantification of Phi29 dependent C-circles from one representative experiment in (**i**). The level of ^{32}P incorporation in the Phi29 negative control samples was subtracted from the samples that contained the Phi29 DNA polymerase. **k**. Representative metaphase image showing MiDAS in *Rap1*$^{-/-}$ MEFs expressing TRF2$^{\Delta B}$. EdU labelled (green) telomeres were detected using PNA probe (red), DAPI used to stain nuclei (blue). EdU signal in a single (white arrowheads) or both chromatids (orange arrowhead). **l**. Quantification of percent EdU positive telomeres in (**k**). Data presents the mean of two experiments ± SD from 30 metaphase with each sample analyzed per experiment. *P=0.0274 by two-sided unpaired t-test.

a

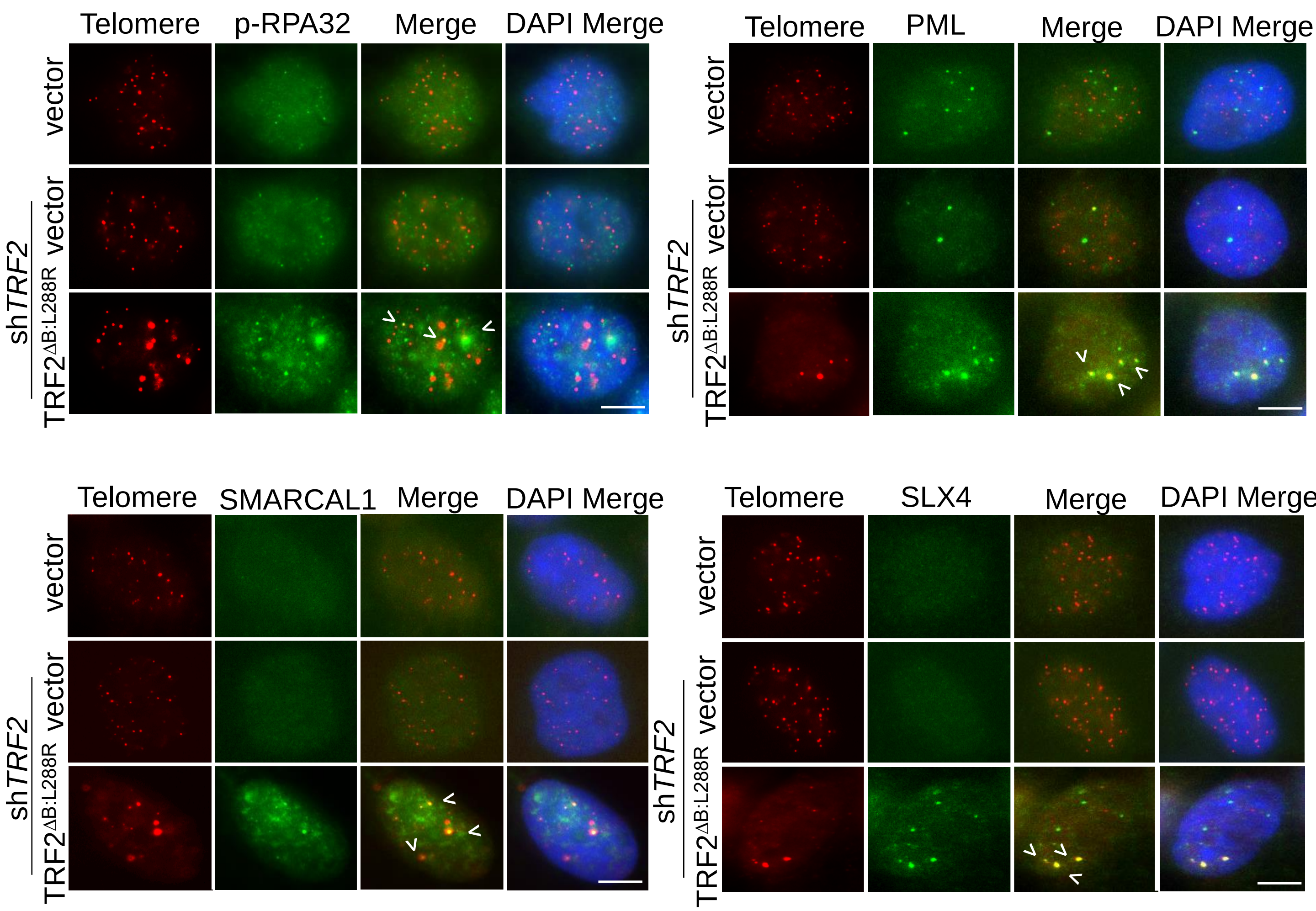

Telomere
p-RPA32
Merge
DAPI Merge
Telomere
PML
Merge
DAPI Merge
vector
shTRF2
vector
TRF2ΔB:L288R
Telomere
SMARCAL1
Merge
DAPI Merge
Telomere
SLX4
Merge
DAPI Merge

b

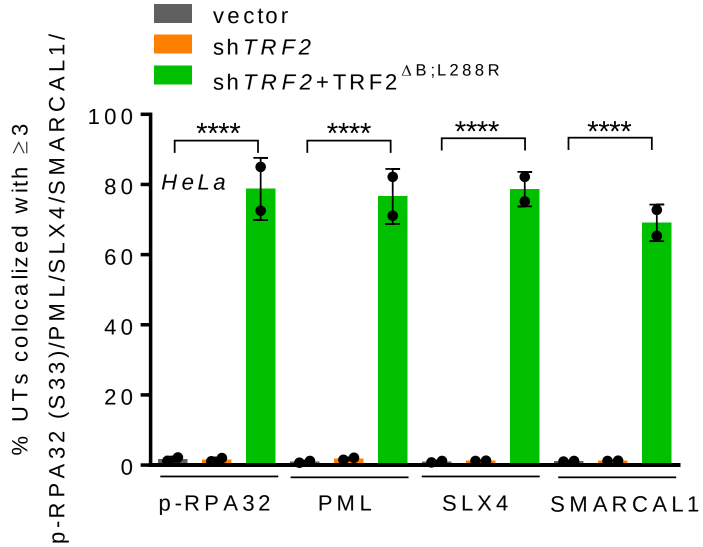

vector
shTRF2
shTRF2+TRF2ΔB;L288R
% UTs colocalized with ≥3
p-RPA32 (S33)/PML/SLX4/SMARCAL1/
HeLa
****
0
20
40
60
80
100
p-RPA32
PML
SLX4
SMARCAL1

c

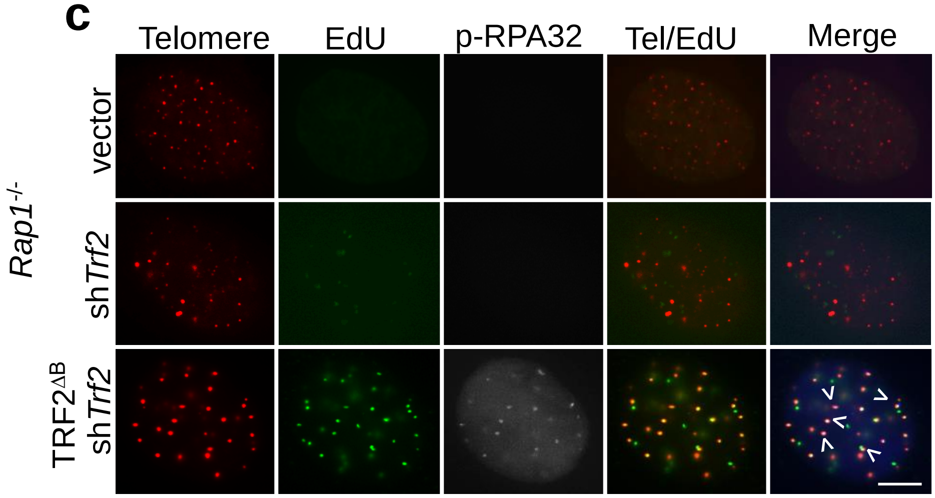

Telomere
EdU
p-RPA32
Tel/EdU
Merge
Rap1-/-
vector
shTrf2
TRF2ΔB
shTrf2

d

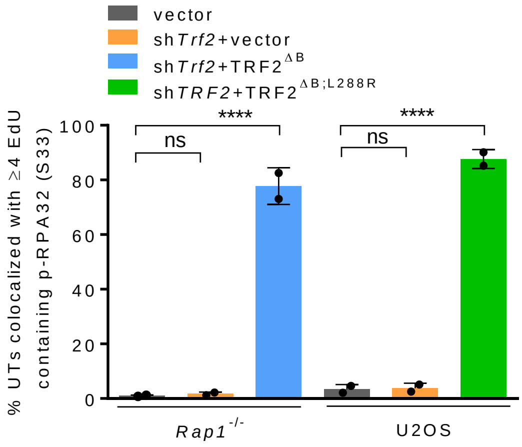

vector
shTrf2+vector
shTrf2+TRF2ΔB
shTRF2+TRF2ΔB;L288R
% UTs colocalized with ≥4 EdU containing p-RPA32 (S33)
100
80
60
40
20
0
ns
****
ns
****
Rap1-/-
U2OS

e

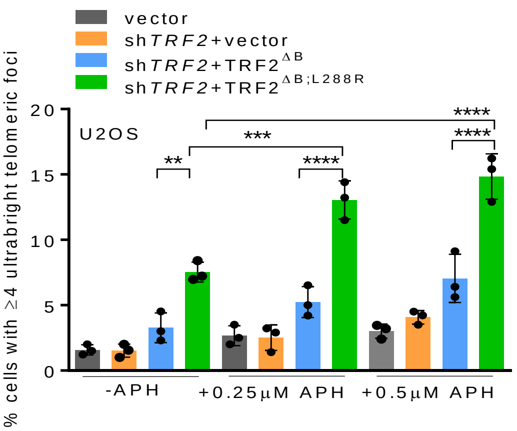

vector
shTRF2+vector
shTRF2+TRF2ΔB
shTRF2+TRF2ΔB;L288R
% cells with ≥4 ultrabright telomeric foci
20
15
10
5
0
U2OS
**
***
****
****
****
-APH
+0.25μM APH
+0.5μM APH

f

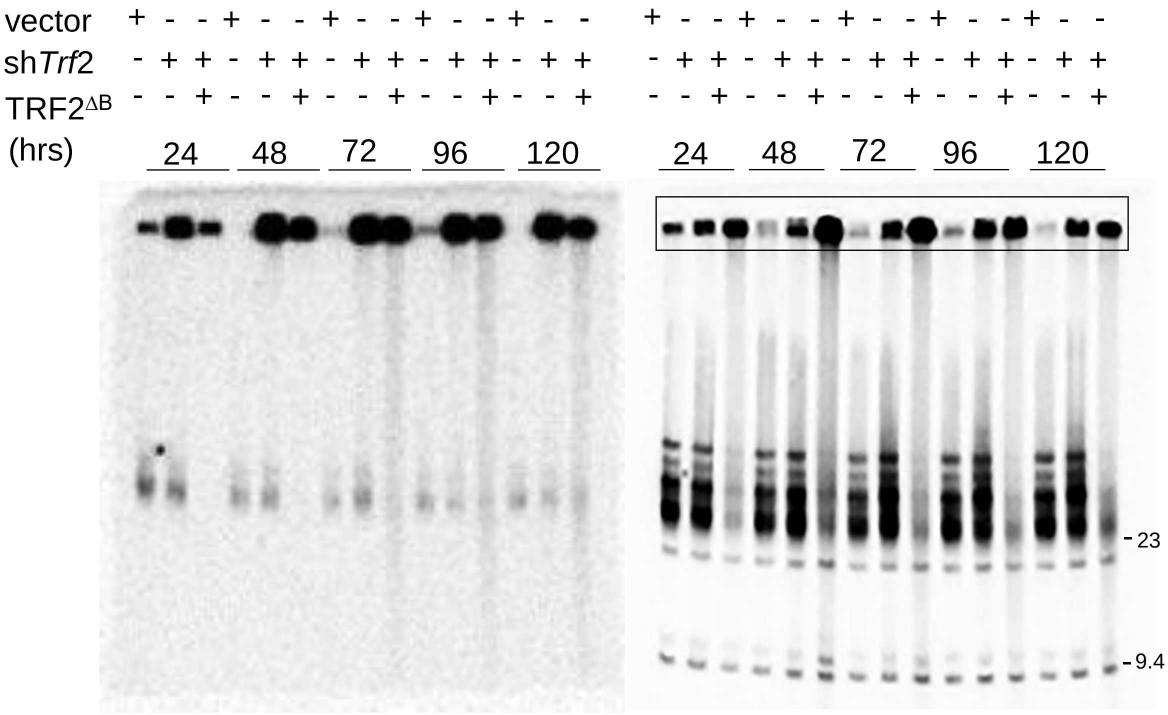

vector
shTrf2
TRF2ΔB
(hrs)
24
48
72
96
120
24
48
72
96
120
23
9.4

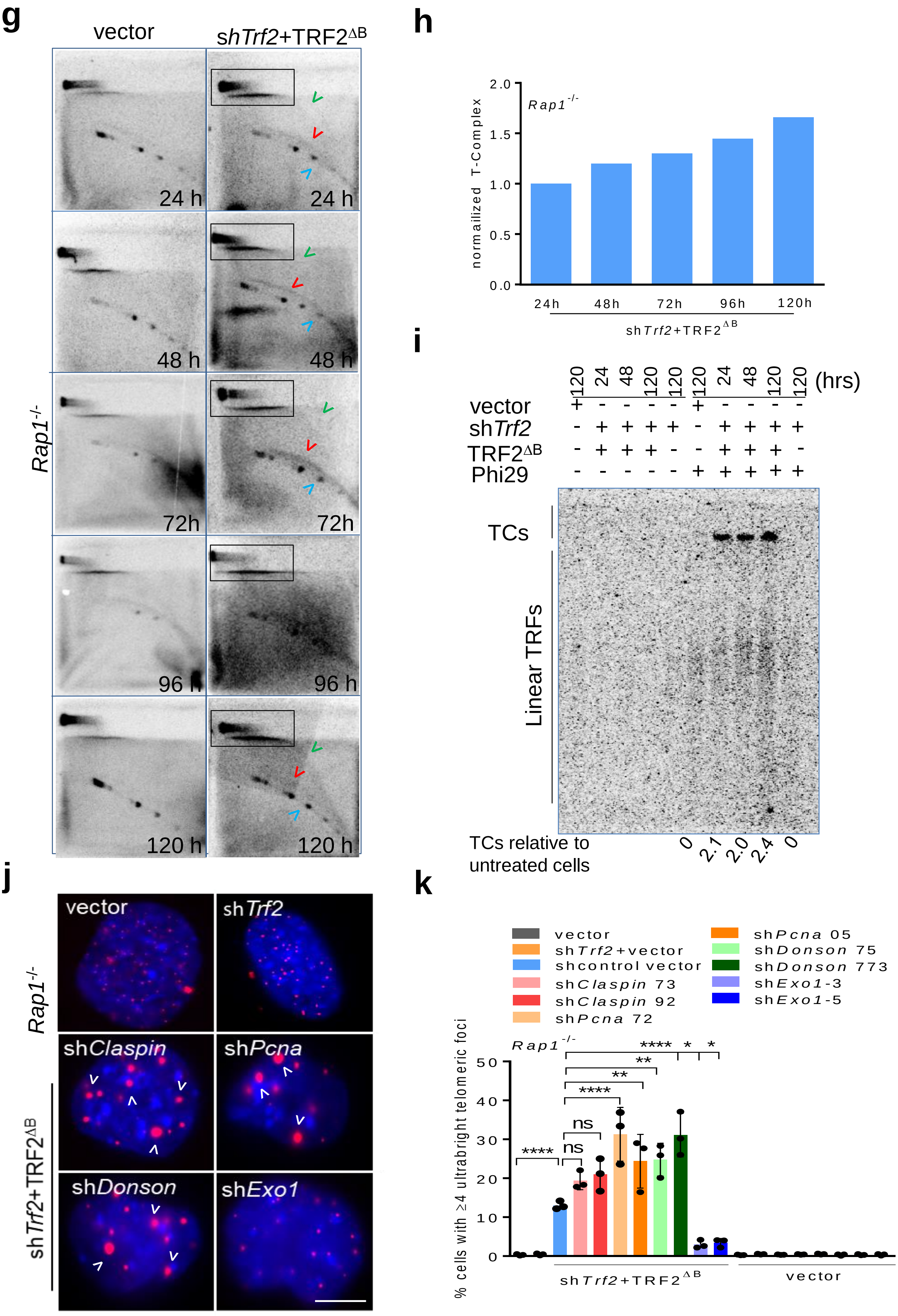
g
vector
shTrf2+TRF2ΔB
Rap1-/-
24 h
48 h
72h
96 h
120 h
h
Rap1-/-
normalized T-Complex
2.0
1.5
1.0
0.5
0.0
24h
48h
72h
96h
120h
shTrf2+TRF2ΔB
i
+120 24 48 120 120 +120 24 48 120 120 (hrs)
vector + - - - - + - - - -
shTrf2 - + + + + - + + + +
TRF2ΔB - + + + - - + + + -
Phi29 - - - - - + + + + +
TCs
Linear TRFs
TCs relative to untreated cells
0 2.1 2.0 2.4 0
j
vector
shTrf2
Rap1-/-
shClaspin
shPcna
shDonson
shExo1
shTrf2+TRF2ΔB
k
vector
shTrf2+vector
shcontrol vector
shClaspin 73
shClaspin 92
shPcna 72
shPcna 05
shDonson 75
shDonson 773
shExo1-3
shExo1-5
Rap1-/-
% cells with ≥4 ultrabright telomeric foci
50
40
30
20
10
0
****
ns
ns
****
**
**
****
*
*
shTrf2+TRF2ΔB
vector

Supplementary Fig. 4 a. Representative IF-FISH showing UTs colocalized with p-RPA32 (S33), PML, SLX4 and SMARCAL1 foci in HeLa cells. White arrowheads point to UTs co-localized to the indicated proteins. Scale bars: 5µm. **b**. Quantification of percent UTs colocalized to HDR factors in HeLa cells expressing $TRF2^{\Delta B;L288R}$. Two independent experiments shown as ± SD from a minimum 150 nuclei analyzed per experiment. ****P<0.0001 by one-way ANOVA. **c**. Representative IF-FISH images showing the localization of telomeres (red) with EdU (green) containing p-RPA32 (S33) (blue) in *Rap1*$^{-/-}$ MEFs expressing indicated constructs. Scale bars: 5µm. **d.** Quantification of percent telomeres colocalized with EdU containing p-RPA32 (S33) in (**c**). Data represents the mean of two independent experiments ± SD from a minimum 200 nuclei analyzed per experiment. ****P<0.0001 by one-way ANOVA. ns: non-significant. **e**. Quantification of UT frequencies in U2OS cells expressing the indicated DNA constructs and with +/– 0.25µM /0.5µM aphidicolin (APH) treatment. Data represents the mean of three independent experiments ± SD from a minimum 300 nuclei analyzed per experiment. ***P*=0.0033, ****P*=0.0001, *****P*<0.0001 by one-way ANOVA. **f**. In-gel single strand G overhang (native, left panel) and total telomere (denatured, right panel) analysis in *Rap1*$^{-/-}$ MEFs expressing the indicated cDNA constructs at the indicated time point. Normalized telomeric DNA trapped in the well (outlined by the box) at indicated time point quantified**.** Signal intensities in vector was set at 100% after normalizing with sub telomeres signals that served as an internal loading control, see Fig. 4f for quantification. **g**. T-complex and T-circle analysis using two-dimensional (2D) gel electrophoresis from genomic DNA isolated from *Rap1*$^{-/-}$ MEFs expressing $TRF2^{\Delta B}$ at indicated time point. T-complex (outlined by the box), TCs (green arrowheads), ss-G (red arrowheads), ds-TRF (blue arrowheads). **h**. Quantification of T-complex from one representative experiment in (**g**). T complex signal intensities in vector was set at 100%. **i**. T-circle assay showing ECTRs at indicated timepoint in *Rap1*$^{-/-}$ MEFs expressing $TRF2^{\Delta B.}$ Phi29 dependent amplification and linear telomere restriction fragments (TRFs) products of TCs were detected by in-gel hybridization using 32P-labeled-T_2AG_3 telomeric probes. Quantification of TCs relative to untreated Phi29 negative samples from one representative experiment shown. **j**. PNA-FISH of interphase nuclei showing UTs in *Rap1*$^{-/-}$ MEFs expressing indicated sh*RNA* constructs against CPD complex and EXO1. Scale bars: 5µm. **k**. Quantification of UT frequencies in (**j**)**.** Data represents the mean of three independent experiments ± SD from a minimum 300 nuclei analyzed per experiment. **P*=0.0214, ***P*=0.0048, ****P<0.0001 by one-way ANOVA. ns: non-significant.

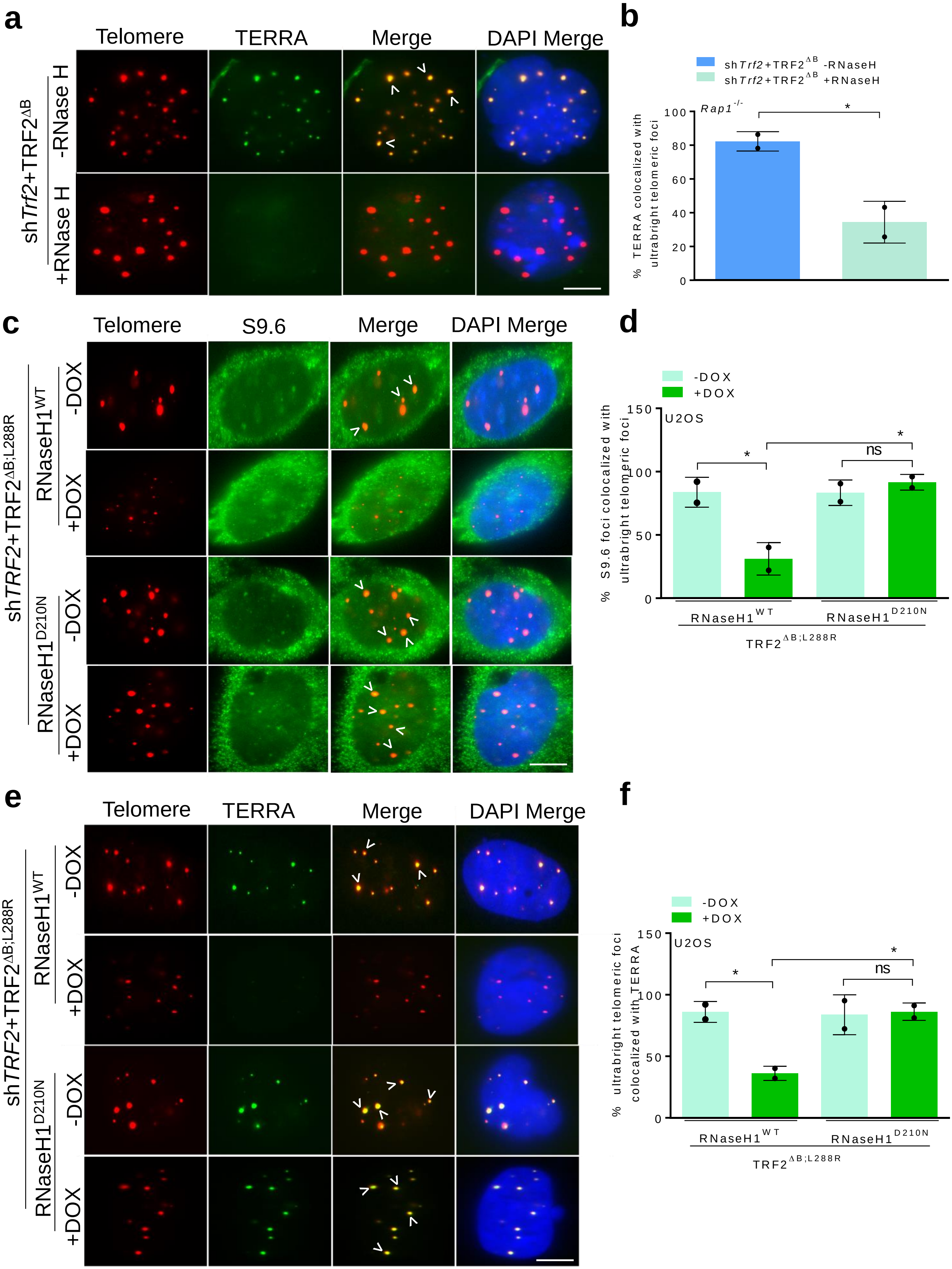
a
Telomere
TERRA
Merge
DAPI Merge
shTrf2+TRF2ΔB
-RNase H
+RNase H
b
shTrf2+TRF2ΔB -RNaseH
shTrf2+TRF2ΔB +RNaseH
Rap1-/-
% TERRA colocalized with ultrabright telomeric foci
c
Telomere
S9.6
Merge
DAPI Merge
shTRF2+TRF2ΔB;L288R
RNaseH1WT
RNaseH1D210N
-DOX
+DOX
d
-DOX
+DOX
U2OS
ns
% S9.6 foci colocalized with ultrabright telomeric foci
RNaseH1WT
RNaseH1D210N
TRF2ΔB;L288R
e
Telomere
TERRA
Merge
DAPI Merge
shTRF2+TRF2ΔB;L288R
RNaseH1WT
RNaseH1D210N
-DOX
+DOX
f
-DOX
+DOX
U2OS
ns
% ultrabright telomeric foci colocalized with TERRA
RNaseH1WT
RNaseH1D210N
TRF2ΔB;L288R

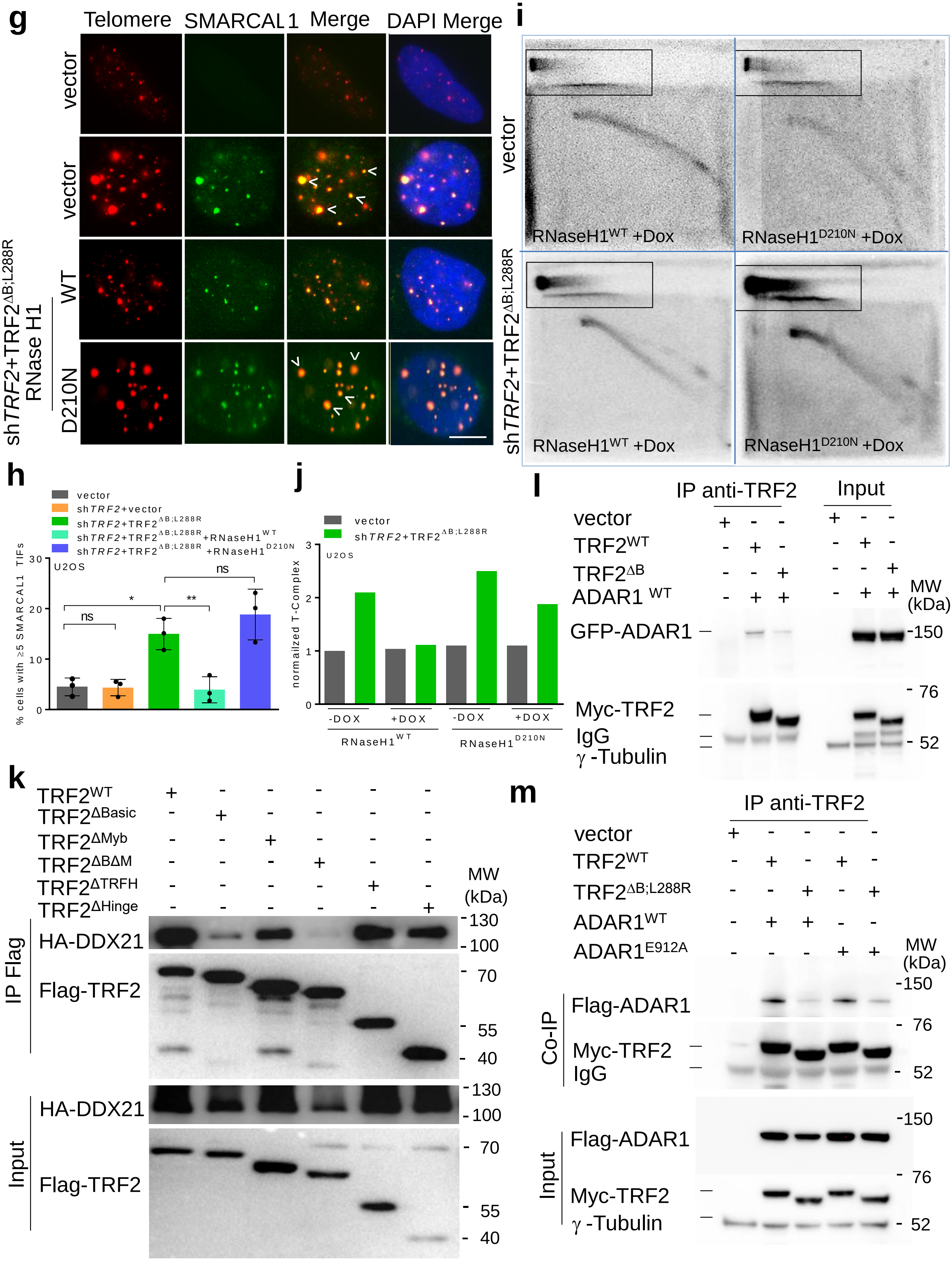
g
Telomere
SMARCAL1
Merge
DAPI Merge
vector
vector
WT
D210N
shTRF2+TRF2ΔB;L288R
RNase H1
i
vector
shTRF2+TRF2ΔB;L288R
RNaseH1WT +Dox
RNaseH1D210N +Dox
h
vector
shTRF2+vector
shTRF2+TRF2ΔB;L288R
shTRF2+TRF2ΔB;L288R+RNaseH1WT
shTRF2+TRF2ΔB;L288R+RNaseH1D210N
U2OS
% cells with ≥5 SMARCAL1 TIFs
ns
*
**
ns
j
vector
shTRF2+TRF2ΔB;L288R
U2OS
normailzed T-Complex
-DOX
+DOX
RNaseH1WT
RNaseH1D210N
l
IP anti-TRF2
Input
vector
TRF2WT
TRF2ΔB
ADAR1 WT
MW (kDa)
GFP-ADAR1
Myc-TRF2
IgG
γ-Tubulin
k
TRF2WT
TRF2ΔBasic
TRF2ΔMyb
TRF2ΔBΔM
TRF2ΔTRFH
TRF2ΔHinge
MW (kDa)
IP Flag
HA-DDX21
Flag-TRF2
Input
HA-DDX21
Flag-TRF2
m
IP anti-TRF2
vector
TRF2WT
TRF2ΔB;L288R
ADAR1WT
ADAR1E912A
MW (kDa)
Co-IP
Flag-ADAR1
Myc-TRF2
IgG
Input
Flag-ADAR1
Myc-TRF2
γ-Tubulin

Supplementary Fig. 5 a. Representative images of IF-FISH from two independent experiments showing the loss of TERRA signal (green) at UTs after incubating the fixed cells with 5U RNaseH for 2h. Scale bars: 5µm. **b**. Quantification of TERRA foci in (**a**). The mean of two independent experiments ± SD from a minimum 250 nuclei analyzed per experiment. *P=0.0384 by two-sided unpaired t-test. **c**. Representative IF-FISH showing the specificity of S9.6 antibody (green) recognizing R-loops at UTs (red) in doxycycline inducible RNaseH1^{WT} or catalytic dead RNaseH1^{D210N} mutant in U2OS cells. S9.6 signal in the cytoplasm is an artifact. Scale bars: 5µm. **d**. Quantification of S9.6 staining in (**c**). The mean of two independent experiments shown as ± SD from a minimum 250 nuclei analyzed per experiment. *P=0.0252 by one-way ANOVA. ns: non-significant. **e**. IF-FISH showing the expression of TERRA (green) in doxycycline inducible RNaseH1^{WT} or catalytic dead RNaseH1^{D210N} mutant in U2OS cells expressing indicated protein. Scale bars: 5µm. **f**. Quantification of TERRA in (**e**). The mean of two independent experiments shown as ± SD from a minimum 200 nuclei analyzed per experiment. **P*=0272 by one-way ANOVA. ns: non-significant. **g**. Representative images of IF-FISH showing UTs co-localized to SMARCAL1 (green) in U2OS cells expressing TRF2$^{\Delta B;L288R}$ in the presence of RNaseH1^{WT} or the catalytic dead RNaseH1^{D210N} mutant. Scale bars: 5µm. **h**. Quantification of percent cells containing ≥5 SMARCALl foci co-localizing with UTs in (**g**). The mean of three independent experiments shown as ± SD from a minimum 350 nuclei analyzed per experiment. **P*=0130, ***P*=0.0092 by one-way ANOVA. ns: non-significant. **i**. T-complex analysis using two-dimensional (2D) gel electrophoresis from genomic DNA isolated from doxycycline inducible RNaseH1^{WT} or catalytic dead RNaseH1^{D210N} mutant in U2OS. T-complex (outlined by the box). **j**. Quantification of T-complex signal intensities in vector was set at 100% in one representative experiment. **k**. Co-IP with Flag antibody with lysates from 293T cells expressing indicated proteins showing that the basic domain of TRF2 is essential to interact with DDX21. The blot shown is the representative of two independent experiments. **l**. Co-IP experiments with TRF2 antibody with lysates from U2OS cells expressing GFP-ADAR1p110^{WT} with Myc-TRF2^{WT} or Myc-TRF2$^{\Delta B}$ showing that basic domain of TRF2 is essential to interact with ADAR1. γ-Tubulin served as loading control. The blot shown is the representative of two independent experiments. **m**. Co-IP experiments with TRF2 antibody with lysates from U2OS cells expressing Myc-TRF2$^{\Delta B;L288R}$ with Flag-ADAR1^{WT} or Flag-ADAR1^{E912A} showing the interaction of TRF2 with ADAR1p110 is independent of ADAR1's catalytic activity. γ-Tubulin served as loading control. The blot shown is the representative of three independent experiments.

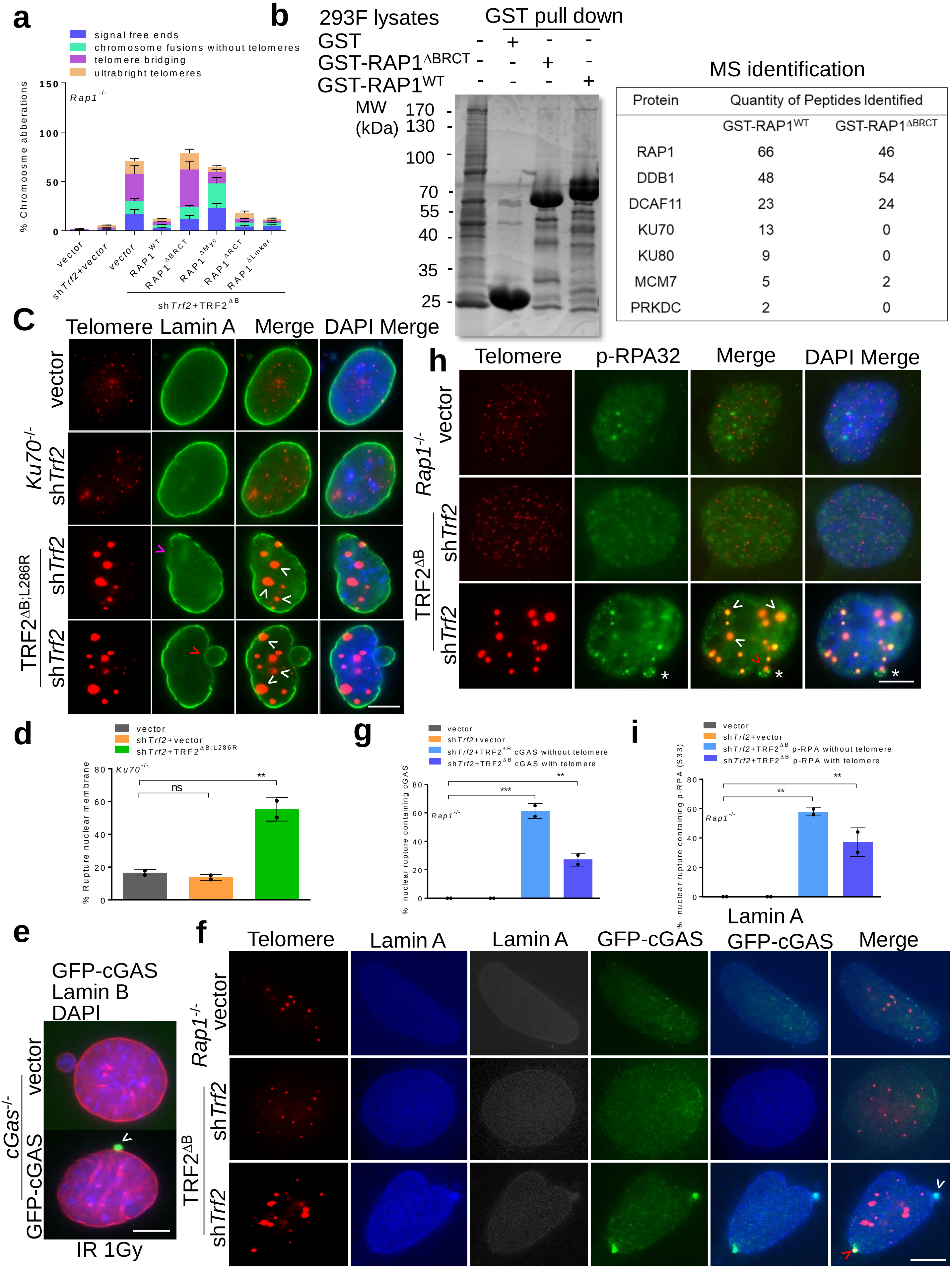
a
signal free ends
chromosome fusions without telomeres
telomere bridging
ultrabright telomeres
% Chromoosme abberations
Rap1-/-
150
100
50
0
vector
shTrf2+vector
vector
RAP1 WT
RAP1 ΔBRCT
RAP1 ΔMyc
RAP1 ΔRCT
RAP1 ΔLinker
shTrf2+TRF2ΔB
b
293F lysates
GST pull down
GST - + - -
GST-RAP1ΔBRCT - - + -
GST-RAP1WT - - - +
MW (kDa)
170
130
100
70
55
40
35
25
MS identification
Protein | Quantity of Peptides Identified
GST-RAP1WT | GST-RAP1ΔBRCT
RAP1 66 46
DDB1 48 54
DCAF11 23 24
KU70 13 0
KU80 9 0
MCM7 5 2
PRKDC 2 0
c
Telomere
Lamin A
Merge
DAPI Merge
Ku70-/-
vector
shTrf2
TRF2ΔB;L286R
shTrf2
shTrf2
h
Telomere
p-RPA32
Merge
DAPI Merge
Rap1-/-
vector
TRF2ΔB
shTrf2
shTrf2
d
vector
shTrf2+vector
shTrf2+TRF2ΔB;L286R
% Rupture nuclear membrane
Ku70-/-
ns
**
g
vector
shTrf2+vector
shTrf2+TRF2ΔB cGAS without telomere
shTrf2+TRF2ΔB cGAS with telomere
% nuclear rupture containing cGAS
Rap1-/-
***
**
i
vector
shTrf2+vector
shTrf2+TRF2ΔB p-RPA without telomere
shTrf2+TRF2ΔB p-RPA with telomere
% nuclear rupture containing p-RPA (S33)
Rap1-/-
**
**
e
GFP-cGAS
Lamin B
DAPI
cGas-/-
vector
GFP-cGAS
IR 1Gy
f
Telomere
Lamin A
Lamin A
GFP-cGAS
Lamin A GFP-cGAS
Merge
Rap1-/-
vector
TRF2ΔB
shTrf2
shTrf2

Supplementary Fig. 6 a. Quantification of signal free-ends, chromosome fusions without telomeres, telomere bridging, UTs in metaphase spread of *Rap1*$^{-/-}$ MEFs expressing Flag tagged TRF2$^{\Delta B}$-RAP1 fusion proteins. The mean values of three independent experiments ± SD are shown, at least 30 metaphases were analyzed per experiment. **b**. GST pull-down in 293F lysate followed by mass spectrometry identification using purified GST-RAP1 WT and GST-RAP1$^{\Delta BRCT}$. The blot shown is the representative of two independent experiments. **c**. IF-FISH showing holes in the NE in sh*Trf2* treated *Ku70*$^{-/-}$ MEFs expressing TRF2$^{\Delta B;L286R}$, characterized by discontinuous and reduced lamin A staining (pink, red arrowhead points to large hole). These cells display robust possessing UTs (white arrowheads) not detected in *Ku70*$^{-/-}$ MEFs treated only with sh*Trf2*. Scale bars: 5μm. **d**. Quantification of nuclear rupture in (**c**). Data shown as the mean of two independent experiments ± SD from a minimum 250 nuclei analyzed per experiment. ***P*=0.0066 by one-way ANOVA. ns: non-significant. **e.** Representative positive control from two independent experiments showing GFP-cGAS (green) reconstituted in *cGas*$^{-/-}$ MEFs localized to NE rupture site (white arrowhead) after 1 Gy irradiation. Lamin B1 (red) stains nuclear envelope. Scale bars: 5μm. **f**. Representative IF-FISH from *Rap1*$^{-/-}$ MEFs showing altered lamin A (blue) promote GFP–cGAS (green) localization to NE rupture sites with indicated treatment. cGAS localization at NE rupture sites with (red arrowhead) and without telomere (white arrowhead). Scale bars: 5μm. **g**. Quantification of GFP-cGAS at NE rupture sites in (**f**). Data shown as the mean of two independent experiments ± SD from a minimum 150 nuclei analyzed per experiment. ***P*=0.0051, ****P*=0.0002, by one-way ANOVA. **h**. Representative IF-FISH showing that p-RPA32 (S33) foci (green) at NE rupture sites (*) containing telomere (red arrowhead) in *Rap1*$^{-/-}$ MEFs expressing indicated proteins. p-RPA32 (S33) foci colocalized with UTs (white arrowheads). Scale bars: 5μm. **i**. Quantification of NE rupture sites containing p-RPA32 (S33) foci with or without telomere in (**h**). Data shown as the mean of two independent experiments ± SD from a minimum 200 nuclei analyzed per experiment. ***P*=0.0012 by one-way ANOVA.

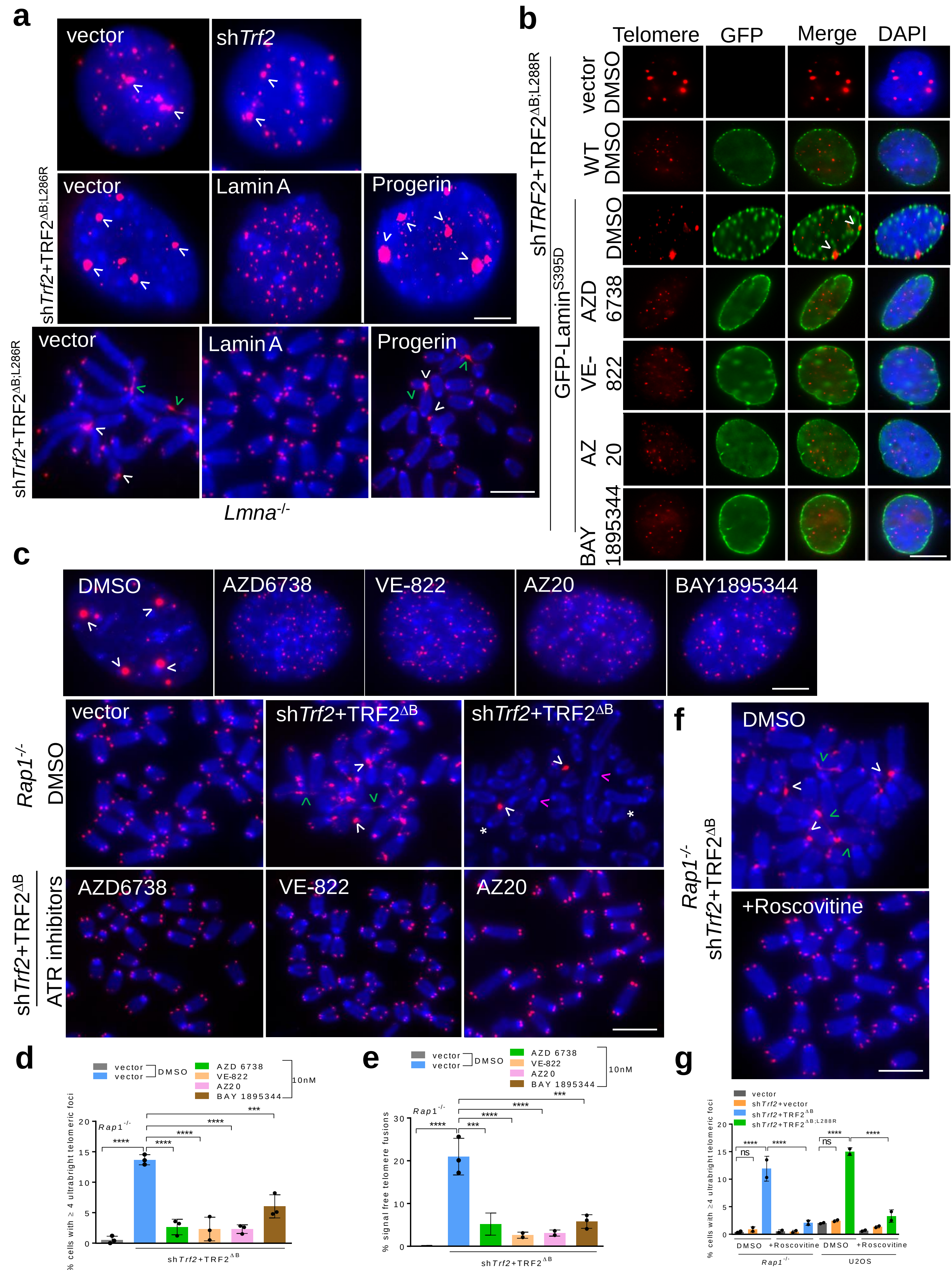
a
vector
shTrf2
shTrf2+TRF2ΔB;L286R
vector
Lamin A
Progerin
shTrf2+TRF2ΔB;L286R
vector
Lamin A
Progerin
Lmna-/-
b
Telomere
GFP
Merge
DAPI
shTRF2+TRF2ΔB;L288R
vector DMSO
WT DMSO
GFP-LaminS395D
DMSO
AZD 6738
VE-822
AZ 20
BAY 1895344
c
DMSO
AZD6738
VE-822
AZ20
BAY1895344
Rap1-/-
DMSO
vector
shTrf2+TRF2ΔB
shTrf2+TRF2ΔB
shTrf2+TRF2ΔB
ATR inhibitors
AZD6738
VE-822
AZ20
f
Rap1-/-
shTrf2+TRF2ΔB
DMSO
+Roscovitine
d
vector
vector
DMSO
AZD 6738
VE-822
AZ20
BAY 1895344
10nM
Rap1-/-
% cells with ≥ 4 ultrabright telomeric foci
shTrf2+TRF2ΔB
e
vector
vector
DMSO
AZD 6738
VE-822
AZ20
BAY 1895344
10nM
Rap1-/-
% signal free telomere fusions
shTrf2+TRF2ΔB
g
vector
shTrf2+vector
shTrf2+TRF2ΔB
shTrf2+TRF2ΔB;L288R
% cells with ≥4 ultrabright telomeric foci
ns
ns
DMSO
+Roscovitine
DMSO
+Roscovitine
Rap1-/-
U2OS

Supplementary Fig. 7 a. Representative PNA-FISH of interphase nuclei and metaphases from three independent experiments showing the formation of CCCTAA-positive filaments (green arrowheads) and UTs (white arrowheads) in *Lmna*$^{-/-}$ MEFs treated with sh*Trf2* +TRF2$^{\Delta B;L286R}$. Scale bars: 5µm. **b**. Representative IF-FISH images from two independent experiments showing the impact ATR inhibitor in the generation of UTs in U2OS cells expressing GFP-lamin^{S395D} (green) and TRF2$^{\Delta B;L288R}$. Scale bars: 5µm. **c**. PNA-FISH on interphase nuclei and metaphases showing the formation of CCCTAA-positive filaments (green arrowheads), UTs (white arrowheads), signal free ends (*) and fused chromosome without telomere at the fusion site (pink arrowheads) in *Rap1*$^{-/-}$ MEFs treated with sh*Trf2*+TRF2$^{\Delta B}$ in the absence and presence of ATR inhibitors. Scale bars: 5µm. **d.** Quantification of UT frequencies in (**c**). Data represents the mean of three independent experiments ± SD from a minimum 300 nuclei analyzed per experiment. ***P=0.0001, ****P<0.0001 by one-way ANOVA. **e.** Quantification of signal free ends in (**c**). Data represents the mean of three independent experiments ± SD from a minimum 300 nuclei analyzed per experiment. ***P=0.0001, ****P<0.0001 by one-way ANOVA. **f**. PNA-FISH on metaphase spread showing CCCTAA-positive filaments (green arrowheads) and UTs (white arrowheads) in *Rap1*$^{-/-}$ MEFs treated with sh*Trf2*+TRF2$^{\Delta B}$ in the absence and presence of 5.0 µM Roscovitine. Scale bars: 15µm. **g**. Quantification of UTs in the interphase nuclei of *Rap1*$^{-/-}$ MEFs and U2OS cells treated with DMSO or 5.0 µM Roscovitine. Data represents the mean of two independent experiments ± SD from a minimum 200 nuclei analyzed per experiment. ****P<0.0001 by one-way ANOVA. ns: non-significant.
