# Supplementary material for: Homology directed telomere clustering, ultrabright telomere formation and nuclear envelope rupture in cells lacking TRF2B and RAP1
Source: Nat Commun. 2023 Apr 14;14:2144. doi: 10.1038/s41467-023-37761-w (PMC10104862; doi:10.1038/s41467-023-37761-w)
Supplement: Supplementary file 3 — Reporting Summary [file 41467_2023_37761_MOESM3_ESM.pdf]

nature portfolio

Corresponding author(s): Rekha Rai, Sandy Chang

Last updated by author(s): 3-15-23

Reporting Summary

Nature Portfolio wishes to improve the reproducibility of the work that we publish. This form provides structure for consistency and transparency in reporting. For further information on Nature Portfolio policies, see our Editorial Policies and the Editorial Policy Checklist.

Statistics

For all statistical analyses, confirm that the following items are present in the figure legend, table legend, main text, or Methods section.

n/a	Confirmed	
☐	☒	The exact sample size (*n*) for each experimental group/condition, given as a discrete number and unit of measurement
☐	☒	A statement on whether measurements were taken from distinct samples or whether the same sample was measured repeatedly
☐	☒	The statistical test(s) used AND whether they are one- or two-sided<br>*Only common tests should be described solely by name; describe more complex techniques in the Methods section.*
☐	☒	A description of all covariates tested
☐	☒	A description of any assumptions or corrections, such as tests of normality and adjustment for multiple comparisons
☐	☒	A full description of the statistical parameters including central tendency (e.g. means) or other basic estimates (e.g. regression coefficient) AND variation (e.g. standard deviation) or associated estimates of uncertainty (e.g. confidence intervals)
☐	☒	For null hypothesis testing, the test statistic (e.g. *F*, *t*, *r*) with confidence intervals, effect sizes, degrees of freedom and *P* value noted<br>*Give P values as exact values whenever suitable.*
☒	☐	For Bayesian analysis, information on the choice of priors and Markov chain Monte Carlo settings
☒	☐	For hierarchical and complex designs, identification of the appropriate level for tests and full reporting of outcomes
☒	☐	Estimates of effect sizes (e.g. Cohen's *d*, Pearson's *r*), indicating how they were calculated

Our web collection on statistics for biologists contains articles on many of the points above.

Software and code

Policy information about availability of computer code

Data collection	Software NIS-Elements BR (Nikon) v3.22.11
Data analysis	ImageJ v1.51k (NIH)<br>GraphPad Prism v7.01<br>ImageQuantTL (GE)<br>Image Lab Touch Software Version 2.3.0.07 (BIO-RAD)

For manuscripts utilizing custom algorithms or software that are central to the research but not yet described in published literature, software must be made available to editors and reviewers. We strongly encourage code deposition in a community repository (e.g. GitHub). See the Nature Portfolio guidelines for submitting code & software for further information.

Data

Policy information about availability of data

All manuscripts must include a data availability statement. This statement should provide the following information, where applicable:

- Accession codes, unique identifiers, or web links for publicly available datasets
- A description of any restrictions on data availability
- For clinical datasets or third party data, please ensure that the statement adheres to our policy

The authors declare that all other data supporting the findings of this study are available within the paper and its supplementary information files.
phospho ELM, a database of S/T/Y phosphorylation sites is available at http://phospho.elm.eu.org/

Human research participants

Policy information about studies involving human research participants and Sex and Gender in Research.

Reporting on sex and gender	N/A
Population characteristics	N/A
Recruitment	N/A
Ethics oversight	N/A

Note that full information on the approval of the study protocol must also be provided in the manuscript.

Field-specific reporting

Please select the one below that is the best fit for your research. If you are not sure, read the appropriate sections before making your selection.

☒ Life sciences ☐ Behavioural & social sciences ☐ Ecological, evolutionary & environmental sciences

For a reference copy of the document with all sections, see nature.com/documents/nr-reporting-summary-flat.pdf

Life sciences study design

All studies must disclose on these points even when the disclosure is negative.

Sample size	No statistical tests were performed to predetermine the sample sizes. Sample sizes were chosen based on previous publications and a sufficient number of replicates was performed to allow calculations of statistical significance.
Data exclusions	No data were excluded from the analysis.
Replication	All the experiments were successfully replicated either two or three times. Details on the number of replicates for each experiment can be found in the associated figure legend. When possible, alternative methods were used to confirm the results.
Randomization	Allocation of samples into the experimental groups was random and the experiments were carried out in parallel.
Blinding	Investigators were blinded to group allocation during both data collection and data analysis for all the experiments involving fluorescence microscopy. Data interpretation of other experiments was based on appropriate controls.

Reporting for specific materials, systems and methods

We require information from authors about some types of materials, experimental systems and methods used in many studies. Here, indicate whether each material, system or method listed is relevant to your study. If you are not sure if a list item applies to your research, read the appropriate section before selecting a response.

Materials & experimental systems

n/a	Involved in the study
☐	☒ Antibodies
☐	☒ Eukaryotic cell lines
☒	☐ Palaeontology and archaeology
☒	☐ Animals and other organisms
☒	☐ Clinical data
☒	☐ Dual use research of concern

Methods

n/a	Involved in the study
☒	☐ ChIP-seq
☒	☐ Flow cytometry
☒	☐ MRI-based neuroimaging

Antibodies

Antibodies used

Primary Antibodies:
CHK2 mouse monoclonal antibody (BD Biosciences), cat#611570, lot 9122675, Dilution 1:2000
RAD51 rabbit polyclonal antibody (Santacruz), cat #sc-8349, clone H-92, lot J2014, Dilution 1:1000
RAD52 mouse monoclonal antibody (Santacruz), cat#sc-365341, clone F7, lot H1518, Dilution 1:1000
Phosphorylated RPA32 (S33) rabbit polyclonal antibody (Bethyl), cat #A300-246A, lot 8, Dilution 1:1000
Phosphorylated CHK1 (S345) rabbit polyclonal antibody (Cell Signaling Technology), cat #sc-2348, clone 133D3, lot 18, Dilution1:1,000
SLX4 rabbit polyclonal antibody (Santacruz), cat# sc-135225, clone H-39, lot F04, Dilution 1:1000 dilution
PML rabbit polyclonal antibody (Santacruz), cat #sc-5621, clone H-238, lot L2806, Dilution 1:1000
Lamin A/C mouse monoclonal antibody (Santacruz), cat #sc-376248, clone E-1, lot G2718, Dilution 1:1000 dilution)
DNA-RNA Hybrid (S9.6) mouse monoclonal antibody (Kerafast), cat #ENH001, clone S.6, lot 200826, Dilution 1:1000 dilution
SMARCAL1 mouse monoclonal antibody (Santa Cruz), cat #sc-376377, clone A-2, lot C1218. Dilution 1:000
TRF2 mouse monoclonal antibody (Millipore), cat #05-521, clone 4A794, lot 3250331. Dilution 1:1000
cGAS rabbit polyclonal antibody (Cell Signaling Technology), cat #15102, clone D1D3G, lot 4, 1:1000 dilution
POLD1 rabbit polyclonal antibody (Bethy), cat#A304-005A, dilution 1;1000
GFP mouse monoclonal antibody (Santacruz) cat#sc-9996, clone B-2, lot G1118, Dilution 1:2000
HA mouse monoclonal antibody (Sigma), cat#H3663, clone HA-7, lot 038m4810v, Dilution 1:2000
FLAG M2 mouse monoclonal antibody (Sigma), cat #F3165, clone M2, lot SLBT6752. Dilution 1:2000
Myc mouse monoclonal antibody (Millipore), cat #05-724, clone 4A6, lot 3095953. Dilution 1:2000
Gamma-Tubulin mouse monoclonal antibody (Sigma), cat# T6557, clone GTU-88, lot 049M4786V. Dilution 1:5000
BrdU mouse monoclonal antibody (BD Biosciences), cat #347580, clone B44. Dilution 1:1000
ATRX rabbit polyclonal antibody (Abcam), cat #ab97508, Dilution 1:1000)
Anti-Flag M2 affinity gel mouse monoclonal antibody (Sigma), cat #A2220, lot SLBN7830V

Secondary antibodies:
Peroxidase-linked anti-mouse IgG (Amersham), cat #NXA931V, lot 16964893. Dilution 1:5000
Peroxidase-linked anti-rabbit IgG (Amersham), cat #NA934V, lot 16991099. Dilution 1:5000
Alexa Fluor 488 goat anti-mouse (Invitrogen), cat #A11001, lot 2140660. Dilution 1:2000
Alexa Fluor 568 goat anti-mouse (Invitrogen), cat #A11004, lot 927620. Dilution 1:2000
Alexa Fluor 488 goat anti-rabbit (Invitrogen), cat #A11008, lot 2018309. Dilution 1:2000
Alexa Fluor 594 goat anti-rabbit (Invitrogen), cat #A11012, lot 1892265. Dilution 1:2000
Alexa Fluor 350 goat anti-rabbit (invitrogen), cat#A11044, lot 1818229. Dilution 1:2000
Alexa Fluor 350 goat-anti-mouse (Invitrogen) cat# A11045, lot 1812517. Dilution 1:2000

Validation

Information on the validation for the antibodies used can be found in the manufacturer's websites.
CHK2 antibody: https://www.bdbiosciences.com/content/bdb/paths/generate-tds-document.us.611570. Validated for Western blot in mouse. Matsuoka S, Huang M, Elledge SJ. (1999) J Biol Chem. 274(44):31463-31467. Rai R. et al. (2016) Nat Commun. 7:10881.

RAD51 antibody (sc-8349): https://www.scbt.com/p/rad51-antibody-h-92. Validated for immunostaining in human and mouse cell lines. Rai R. et al. (2016) Nature commun. 7: 10881. Rai R. et al. (2019) Cell Rep. 29(11):3708-3725.e5.
Hariharasudhan G. et al. (2022) Nucleic Acids Res. 50(3): 1501–1516. Cox KE. et al. (2016) Cell Rep. 14(5), 9 1032-1040.

RAD52 antibody: https://datasheets.scbt.com/sc-365341. Validated for Western blot and immunostaining in human an mouse cells. Barroso-González, J., et al. (2019) Mol. Cell 76: 11-26.

Phosphorylated RPA32 (S33) antibody (A300-246A): https://www.bethyl.com/product/A300-246A/Phospho. Validated for Western blot in human cells. Several research articles show immunostaining data obtained with this antibody. Silva B. et al. (2019) Nat. Commun. 10: 2253. Wang YH. et al. (2017) Nat. Commun. 8: 2118. Zhang B. et al. (2014) J Biol. Chem. 289(49): 34284-34295. Graziano S. et al. (2021) JBC 297 (5):101301. Rai R. et al. (2019) Cell Rep. 29(11):3708-3725.e5.

Phosphorylated CHK1 (sc-2348):https://www.cellsignal.com/products/primary-antibodies/phospho-chk1-ser345-133d3-rabbit-mab/2348. Validated for Western blotting. Rai R. et al. (2010) EMBO J. 29 (15):2598-610. Rai R. et a., (2016) Nat Commun. 7:10881. Zhao H and Piwnica-Worms H. (2001) Mol Cell Biol. 21:4129-39.

SLX4 antibody (sc-135225): https://www.citeab.com/antibodies/782111-sc-135225-btbd12-h-39. Validated for Western blotting. Several research articles showed immunostaining data obtained with this antibody. Rai R. (2016) Nat Commun. 7:10881. Margalef P. et al. (2018) Cell 172(3): 439–453.e14.

PML antibody (sc-5621): (https://www.scbt.com/p/pml-antibody-h-238. Validated for immunostaining in human and mouse cell lines. Marchesini et al. (2016) Oncogene 35, 1811-1821. Flynn R. et al. (2015) Science 2015 347 (6219):273-7, Mason-Osann E. et al. (2018)

Oncotarget. 9(67): 32868–32880.

Lamin A/C (sc-376248): https://www.scbt.com/p/lamin-a-c-antibody-e-1. Validated for Western blotting and immunostaining. Shah P. et al. Front. Cell Dev. Biol. 10: 3389. Kronenberg-Tenga R. et al. (2021) J Cell Sci 134 (6): jcs256156.

DNA-RNA Hybrid S9.6 antibody (ENH001): https://www.kerafast.com/productgroup/432/anti-dna-rna-hybrid-s96-antibody?ProductID=2082. Validated for immunohistochemistry in human cells. Several research articles showed immunostaining data obtained with this antibody. Bhatia V. et al. (2014) Nature 511(7509):362-5. Chang YC. et al. (2019) Nature Commun. 10:4265. Yang and Zhang. (2022) STAR Protocols 3: 101325.

SMARCAL1 antibody (sc-376377): https://www.scbt.com/p/smarcal1-antibody-a-2. Validated for Western blot and immunostaining of human cell lines and human tissues. Rai R. et al. (2019) Cell Rep. 29(11):3708-3725.e5. Nazeer R. et al. (2019) J Virol. 93(13): e00402-19. Cox KE. et al. (2016) Cell Rep. 14(5), 9 1032-1040.

TRF2 antibody (05-521): https://www.emdmillipore.com/US/en/product/Anti-TRF2-Antibody-clone-4A794,MM_NF-05-521. Validated by immunoblot on RIPA lysate of human Jurkat cells and HeLa nuclear extract. Rai R. et al. (2019) Cell Rep. 29(11):3708-3725.e5. Wakai M. et al. (2014) PloS One 9: e88530. Delbarre E. et al. (2013) Genome Res. 23(3):440-51.

cGAS rabbit polyclonal antibody (15102): https://www.cellsignal.com/products/primary-antibodies/cgas-d1d3g-rabbit-mab/15102 Validated for Western blot and immunostaining in mouse cells. Chen et al., (2020) Sci Adv. 6(42): eabb8941.

POLD1 rabbit polyclonal antibody (A304-005A): https://www.thermofisher.com/antibody/product/PolD1-Antibody-Polyclonal/A304-005A.

GFP (sc-9996): https://www.scbt.com/p/gfp-antibody-b-2?requestFrom=search. This antibody has been used for Immunostaining in COS cells transfected with GFP fusion protein showing cytoplasmic staining. Rai R. et al. (2019) Cell Rep. 29(11):3708-3725.e5.

HA antibody (H3663):https://www.https://www.sigmaaldrich.com/US/en/product/sigma/h3663?context=product# Validated for use in CHIP, IC, IP, WB for the detection of HA Tag. Chen C. et al (2017) Nat Commun 8, 14929, Markus H. et al (2020) Cell 181(2):271-280.e8.Rai R. et al. (2019) Cell Rep. 29(11):3708-3725.e5.

FLAG M2 antibody (F3165): https://www.sigmaaldrich.com/catalog/product/sigma/f3165?lang=en&region=US. Validated for Western blot and immunostaining for the detection of FLAG tag. Rai R. et al. (2019) Cell Rep. 29(11):3708-3725.e5. Sriramachandran AM. et al. (2019) Nat Commun. 10:3678. Doyle SL. et al. (2012) Nat Commun. 3:707.

Myc antibody (05-724): https://www.emdmillipore.com/US/en/product/Anti-Myc-Tag-Antibody-clone-4A6,MM_NF-05-724. Validated for use in ChIP, IC, IF, IP, WB for the detection of Myc Tag. Rai R. et al. (2019) Cell Rep. 29(11):3708-3725.e5. Zhang S. et al. (2015) Nat Neurosci. 18(3):386-92.

Gamma-Tubulin antibody (T6557): https://www.sigmaaldrich.com/catalog/product/sigma/t6557?lang=en&region=US. Validated for Western blot and immunostaining in human cell lines. Rai R. et al. (2019) Cell Rep. 29(11):3708-3725.e5. Rai R. et al. (2017) Mol Cell 65(5): 801–817.e4. Rai R. et al. (2016) Nature Commun. 7: 10881.

BrdU mouse monoclonal antibody (347580): https://www.bdbiosciences.com/us/applications/research/apoptosis/purified-antibodies/purified-mouse-anti-brdu-b44/p/347580. Validated for both flow cytometry and immunofluorescence in human cells. Mukherjee B. et al. (2015) Methods Mol Biol. 1292: 67–75.

ATRX antibody (ab97508): https://www.abcam.com/products/primary-antibodies/atrx-antibody-ab97508.html. Validated for Western blot and immunostaining in human and mouse cell lines. Lovejoy CA et al. (2020) Biol. 18:e3000594.

Anti-Flag M2 affinity gel (A2220): Validated for immunoprecipitation and purification of Flag fusion proteins. Yu Ti Cheng et al.(2011) 108(35), 14694-14699. Nora Nonne et al.(2009) Nucleic acids research, 38(4), e20-e20 .

Eukaryotic cell lines

Policy information about cell lines and Sex and Gender in Research

Cell line source(s)	293T: ATCC (CRL-3216), HeLa: ATCC (CCL-2), U2OS:ATCC (HTB-06), Saos-2 ATCC (HTB-85), IMR-90: ATCC (CCL-186), Ku70+/+, Ku70-/- : Dr. Sandy Chang, Rap1+/+ and Rap1-/- MEFs: Dr. Maria A. Blasco (CNIO Spain), Lmna+/+ and Lmna-/- MEFs: Dr. Susana Gonzalo (St Louis University School of Medicine), Dox inducible RNaseH1WT, RNaseH1D210N U2OS cells: Dr. Lilian Kabeche (Yale University), Nbs1-/- MEFs: Dr. Kenshi Komatsu (Kyoto Japan)
Authentication	These cell lines are routinely used in our lab and we constantly monitor their morphology.
Mycoplasma contamination	All cell lines tested negative for mycoplasma contamination by PCR.
Commonly misidentified lines (See ICLAC register)	No commonly misidentified cell lines were used in this study.
